# 

**Published:** 1875-01

**Authors:** 


CONTENTS:

| | PAGE |
|---|---|
| The Sunday Question | 3 |
| Object of Eating | 6 |
| Nature's Uniformity | 7 |
| Opium and Religion | 7 |
| Health of Great Cities | 8 |
| Milk Diet | 9 |
| To Filter Water | 10 |
| Whiskey Drinking | 11 |
| Substances in the Nose | 12 |
| Take Time by the Forelock | 13 |
| Sanitary Science | 14 |
| House-Building Materials | 15 |
| Painting the Lily | 16 |
| Let Nothing be Wasted | 17 |
| Supplying New Blood | 18 |
| Give us Long Life | 19 |
| Rheumatism | 20 |
| Tenement Houses | 20 |
| Dangers of Travel | 21 |
| Stone or Gravel | 21 |
| The House of Steinway | 23 |
| Distortions of the Feet | 24 |
| Our Home on the Hillside | 26 |
| Infant Mortality | 28 |
| How we Squander Health | 30 |
| Warming Houses | 31 |
| Intemperance | 32 |
| The Teeth | 33 |
| Twenty-Second Year | 34 |
| Caution | 35 |
| Libraries | 36 |
| Removal | 37 |

